# Phylogenetic regionalization of ectoparasites and their hosts using 2 approaches: a case study with fleas and their rodent hosts from Mongolia

**DOI:** 10.1017/S0031182025101212

**Published:** 2026-01

**Authors:** Renan Maestri, Uri Roll, Vasily I. Grabovsky, Georgy I. Shenbrot, Boris R. Krasnov

**Affiliations:** 1 Departamento de Ecologia, Universidade Federal do Rio Grande do Sul, Porto Alegre, Brazil; 2 Mitrani Department of Desert Ecology, Swiss Institute for Dryland Environmental and Energy Research, Jacob Blaustein Institutes for Desert Research, Ben-Gurion University of the Negev, Sede Boqer Campus, Midreshet Ben-Gurion, Israel; 3 French Associates Institute for Agriculture and Biotechnology of Drylands, Jacob Blaustein Institutes for Desert Research, Ben-Gurion University of the Negev, Sede Boqer Campus, Midreshet Ben-Gurion, Israel

**Keywords:** evoregions, hosts, parasites, phyloregions, scale-dependence

## Abstract

We applied 2 methods of phylogenetic regionalization (evoregions and phyloregions) for the distributions of fleas and their rodent hosts across Mongolia. We investigated the congruence between these 2 regionalization schemes and their alignment with physiographic and ecological subdivisions of Mongolia. We identified evoregions and phyloregions for both fleas and hosts. Ancestral regional distributions were reconstructed, and a phylogenetic correspondence analysis identified key contributing lineages. Using the V-measure, we tested for the congruence between (a) evoregions or phyloregions identified for fleas and evoregions or phyloregions, respectively, identified for their hosts and (b) evoregions and phyloregions identified for either fleas or hosts and each of the physiographic/ecological regionalization schemes of Mongolia. Four evoregions and 8 phyloregions were identified for both fleas and hosts, exhibiting distinct spatial patterns. Host-parasite regionalizations demonstrated moderate spatial similarity (V-measure 0.49–0.50), a significantly higher congruence than previously reported at the larger Palearctic scale (0.33). Flea regionalizations exhibited stronger congruence with environmental schemes than did host regionalizations. We concluded that evoregionalization and phyloregionalization capture distinct evolutionary signals, reflecting the role of *in situ* diversification vs. phylogenetic turnover resulting from dispersal. Host-parasite co-regionalization is scale-dependent, with increased congruence at regional scales. Despite adult fleas’ obligate host dependence, their regionalization is not merely a passive reflection of host biogeography but is also profoundly shaped by environmental conditions. These findings emphasize the importance of method choice, scale and eco-evolutionary interactions in shaping complex biogeographic patterns.

## Graphical abstract

**Figure:**
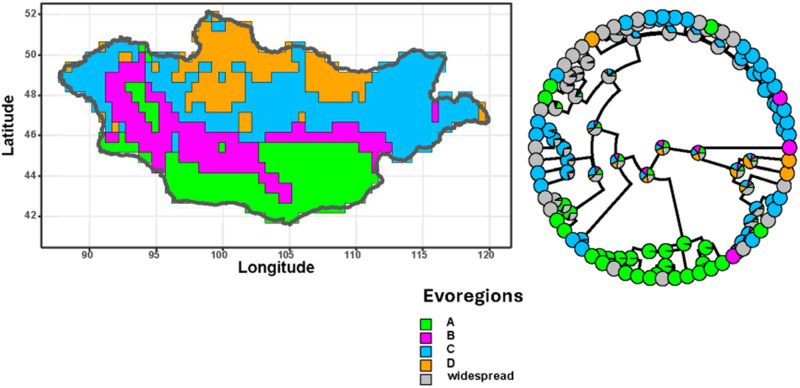

## Introduction

Biogeographic regionalization, the delineation of areas characterized by unique biological assemblages, is a fundamental concept in macroecology and conservation (e.g. Kreft and Jetz, 2010; Vilhena and Antonelli, 2015; Edler et al. 2017). These regions are not arbitrary divisions but rather products of complex historical processes (Morrone, 2018; Rodrigues and Duarte, 2024). Such processes encompass both *in situ* diversification, where lineages evolve and speciate within a given area, and *ex situ* diversification, driven primarily by dispersal and subsequent colonization of new territories (Daru et al. 2016, 2020a; Maestri and Duarte, 2020; Nakamura et al. 2024). Generally, *in situ* diversification tends to shape biogeographic patterns at broader scales, such as global biogeographic realms (e.g. Holt et al. 2013), while *ex situ* processes, particularly dispersal, play a more pronounced role in shaping regionalization within these larger areas (e.g. Gibert et al. 2021).

Traditional regionalization approaches often rely solely on species presence/absence data (e.g. Kreft and Jetz, 2010; Rueda et al. 2013; Leroy et al. 2019, see Denelle et al. 2025 for review), which can overlook the deep evolutionary history that underpins biodiversity patterns. Incorporating phylogenetic information is crucial to understand how evolutionary processes have shaped current distributions and for defining regions based on a shared evolutionary heritage and unique diversification histories (Goldberg et al. 2011; Holt et al. 2013).

Recent methodological advancements have enabled a more nuanced integration of phylogeny into regionalization. Among these, 2 prominent approaches are evoregionalization (sensu Maestri and Duarte, 2020) and phyloregionalization (sensu Daru et al. 2016). Evoregions are delineated based on phylogenetic turnover that specifically accounts for *in situ* diversification. This method incorporates the difference between the numbers of tips descending from internal nodes (i.e. tree imbalance), making it sensitive to diversification patterns. In contrast, phyloregions define areas based on phylogenetic distinctness, quantifying shared evolutionary histories among assemblages, using metrics such as the Simpson phylogenetic beta diversity index, which emphasizes species turnover irrespective of richness differences. The choice of method can be influenced by the spatial scale of interest. Evoregionalization, by directly incorporating aspects of diversification and tree imbalance, is often considered more suitable for delineating macroevolutionary patterns at larger scales, such as biogeographic realms, where deep evolutionary divergences are prominent (Maestri and Duarte, 2020; Krasnov and Shenbrot, 2023; Nakamura et al. 2024). At smaller spatial scales (e.g. regional) where dispersal might be a more dominant driver of biotic exchange, its application needs careful consideration. Phyloregionalization, however, due to its robustness to differences in species richness and its focus on phylogenetic turnover, has proven applicable and informative across a wider range of spatial scales, from global to regional (Daru et al. 2016, 2020a; Ruggiero and Morrone, 2025). However, diversification of any taxon may likely occur not only across large but also across restricted geographic areas. For example, allactagine dipodids have been suggested to have begun diversification in Central Asia. During the wet period of the Lower Pliocene, the eastern and western parts of Central Asia became separated by an environmental barrier, so the diversification of 2 sister allactagine clades (*Allactaga-Allactodipus-Orientallactaga* and *Scarturus-Pygeretmus*) took place independently in the 2 resulting regions (Lebedev et al. 2022). Moreover, the occurrence of multiple species belonging to the same phylogenetic lineage in a given region suggests either that this lineage diversified in that region or that all of them dispersed there from somewhere else, while still conserving their phylogenetic relationships. In other words, phylogenetic relationships of such regionally co-occurring closely-related species may mimic *in situ* diversification even if their real diversification centre is somewhere else. This suggests that evoregionalization might be applied at scales smaller than, for example, the continental or biogeographic realm scale. This, however, has never been done.

Understanding biogeographic patterns for interacting taxa, such as parasites and their hosts, presents a unique challenge and opportunity. A parasite’s spatial distribution is fundamentally dependent on that of its host(s), yet their evolutionary histories might unfold at different rates or be influenced by distinct environmental filters. Our previous work (Maestri et al. 2017, 2020a) explored the relationships between the non-phylogenetic and phylogenetic beta diversities of fleas and their small mammalian hosts in Mongolia without regionalization schemes. In both cases, we found a strong dependence of flea phylogenetic beta diversity on host phylogenetic beta diversity. However, when the evoregionalization of fleas and their hosts was carried out at a much larger scale (across the entire Palearctic), the geographic positions of evoregions for fleas and hosts did not appear to match each other. This discrepancy suggests that relationships between parasite and host phylogenetic beta diversity may not reflect the relationships between the schemes of their phylogenetic regionalizations, although the link between these patterns can be expected (Daru et al. 2016). To further understand this, we here applied both evoregionalization and phyloregionalization approaches for the regionalization of fleas and their rodent hosts within Mongolia.

Specifically, we asked whether (a) evoregionalization conforms to phyloregionalization (or vice versa) for either fleas or hosts; (b) either evoregions or phyloregions for fleas and hosts match each other; and (c) either evoregions or phyloregions for either fleas or hosts conform to physiographic or ecological regions (biomes and ecoregions) of Mongolia. In addition, we asked whether the pattern of congruence between the evoregions of fleas and hosts at a smaller scale (e.g. across Mongolia) differs from that at a larger scale (the entire Palearctic; Krasnov and Shenbrot, 2023). We expected to see this difference between scales because of the scale-dependence of a variety of biogeographic patterns (e.g. Lyons and Willig, 1999; Daru et al. 2020a). We selected Mongolia because it is one of the few regions of the world where the most comprehensive long-term studies of fleas have been carried out. In fact, Mongolia has been surveyed for rodents for almost a century and for fleas for almost 40 years (see sources and references in Maestri et al. 2020a). We focused on fleas harboured by rodent hosts because (a) the sampling effort aimed at other host taxa that harbour fleas (birds, carnivores, ungulates and bats) was much weaker than that for rodents and (b) fleas are the most abundant and diverse on this mammalian taxon (Krasnov, 2008). In addition, Mongolia represents an important ecological transition zone between the Siberian taiga forests, the Altai Mountains and the Gobi Desert.

## Materials and methods

### Flea and rodent distributions across Mongolia

This study involved the re-analysis of data of flea and rodent distributions (including Species Distribution Models) across Mongolia, primarily sourced from our earlier studies (Maestri et al. 2017, 2020a, 2020b). The initial data represented the occurrence of rodents and fleas across 2370 sites evenly distributed throughout Mongolia (see map in Maestri et al. 2017). Mongolia was comprehensively surveyed for both rodents and fleas in the framework of the Central Asiatic Expeditions of the American Museum of Natural History in Mongolia and China, the Mongolian-German Biological Expeditions, Joint Soviet/Russian-Mongolian Complex Biological Expedition and a joint project between the National University of Mongolia (Ulaan Baatar) and the Museum of Southwestern Biology of the University of New Mexico (MSB, Albuquerque, NM, USA). In addition to the data taken from the literature (see references in Maestri et al. 2020a), we used occurrence records from the databases of the Zoological Museum of Moscow State University (Moscow, Russia), the Zoological Museum of the Zoological Institute of the Russian Academy of Sciences (Saint Petersburg, Russia) and the data generously provided by the late Dr M. Kiefer who carried out a comprehensive survey of fleas in Mongolia from 1968 to 1984. We also used published maps of the occurrences of rodent and flea species (Kiefer et al. 1984, 2012; Lebedev et al. 2016). In total, occurrence records for 64 rodent and 66 flea species were compiled from the captures of approximately 20 000 individual rodents and 15 000 individual fleas.

In brief, a distribution model for each species was constructed based on the occurrence records and using environmental data (elevation and 19 variables describing temperature and precipitation gradients taken from the WorldClim 2.0 database, data for 1970–2000; Fick and Hijmans, 2017) employed as 30 arc-second grids (ca. 1-km resolution) (see details in Maestri et al. 2017. The distribution models for fleas were based on environmental variables only, but not on the distribution of their hosts. This is because (a) majority of flea species demonstrate rather loose host specificity and are able not only to exploit several host species in the same locality (e.g. Krasnov, 2008) but also replace 1 host species with another host species across localities within a flea’s geographic range (Shenbrot et al. 2007) and (b) the entire spectrum of host species that a flea is able to utilize is unknown for the majority of flea species. A model for each flea or host species was built with MAXENT 3.4 software (Phillips et al. 2006) using default settings and the MAXENT logistic output. This output provided estimates of relative environmental suitability for a given species (Elith et al. 2011). We added a 2°-buffer to the polygon of the Mongolian territory, converted the buffer to the 30 arc-second grids, and assigned values of 1 inside the buffer and ‘NoData’ outside it. This raster was then used as the mask for clipping environmental variables to the area of interest. The model outputs, representing environmental suitability estimates, ranged from 0 to 1. These values were subsequently transformed to binary data (0 or 1), using a threshold value equal to either (a) the ‘minimal training presence’ for species with less than 25 occurrence points or (b) the ‘maximum training sensitivity plus specificity’ for species with more than 25 occurrence points (Liu et al. 2013). The resulting binary raster grids created for each species were laid onto a map of Mongolia divided into a grid of 0.5° × 0.5° cells using ArcMap 10.3 software. Then, the occurrence records of each species (fleas and rodents separately) were transformed into a grid cell × species matrix, with the presence/absence of each species in each grid cell. Geographic coordinates of each grid cell were taken as the coordinates of its centroid.

Further details on the construction of distribution models and evaluation of model performance can be found in Maestri et al. (2020a). In brief, the performance of each SDM was evaluated by (a) threshold-independent area under the receiver operating characteristic curve (AUC) and (b) threshold-dependent true skill statistic (TSS; Allouche et al. 2006). The AUC values range between 0 and 1, with the value of 1 indicating an ideal good performance. TSS represents sensitivity + specificity – 1 (Allouche et al. 2006) with sensitivity denoting the proportion of predicted observed presences (quantification of omission errors) and specificity denoting the proportion of predicted observed absences (quantification of commission errors) (Shabani et al. 2016). TSS values can range from −1 to 1, with a zero value indicating that a model distribution does not differ from random. For each species, we used randomly selected training data to calculate training AUC and training TSS and randomly selected test data to calculate test AUC and test TSS. This procedure was repeated 30 times. The quality of species distribution models appeared to be relatively high, with mean training and testing AUC values for rodents being 0.914 and 0.872, respectively, and training and testing AUC values for fleas being 0.907 and 0.826, respectively. Mean training and testing TSS values for rodents were 0.737 and 0.679, respectively, and training and testing TSS values for fleas were 0.705 and 0.576, respectively.

### Physiographic and ecological regionalizations of Mongolia

A map of the physiographic regionalization of Mongolia was taken from Dorjgotov (2025). This regionalization divides the territory of Mongolia into either 4 main regions or 12 subregions (see Figs. S1 and S2, respectively, in Appendix 1 of the Supplementary Material). The map was digitized using ArcGIS Pro version 3.53 (ESRI 2025). The ecological regionalization was represented by either 5 main biomes (Fig. S3 of Appendix 1 of the Supplementary Material) or 15 ecoregions (Fig. S4 of Appendix 2 of the Supplementary Material), taken as shapefiles from Dinerstein et al. (2017) and the website https://ecoregions.appspot.com/. We used the World Geodetic System 1984 to produce the 0.5° × 0.5° grid of Mongolia in each of these maps.

### Phylogenies

As a backbone for flea phylogeny, we used the most comprehensive molecular phylogenetic tree available (Zhu et al. 2015), which includes most flea genera, but not species, from our datasets. For genera and species that are not present in Zhu et al.’s (2015) tree, the topology was established based on their morphologically derived taxonomic positions (see Hadfield et al. 2014). We assigned all branch lengths to 1 because no information on branch lengths was available. Then, the tree was ultrametrized using the ‘force.ultrametric’ function of the R package ‘phytools’ (Revell, 2012), implemented in the R Statistical Environment (R Core Team, 2025). Flea species names follow Hastriter and Bossard (2025).

To construct a phylogenetic tree for rodent host species, we took 1000 random subsets for 64 rodent species in our dataset from the 10 000 species-level birth-death tip-dated completed trees for 5911 mammal species of Upham et al. (2019). We constructed a consensus tree using the ‘consensus.edge’ function of the ‘phytools’ package. Then, the resulting tree was ultrametrized (as described above), and the polytomies were resolved using the ‘fix.poly’ function of the R package ‘RRphylo’ (Castiglione et al. 2018). Rodent species names follow Wilson et al. (2017) and Kryštufek and Shenbrot (2022, 2025). It should be noted that the topology of some clades of Upham et al.’s (2019) trees weakly conforms to recently published phylogenetic relationships (e.g. Lebedev et al. 2022). Nevertheless, Upham et al.’s (2019) trees represent the most comprehensive-to-date mammalian phylogenies.

### Data analyses

Prior to analyses, the columns (i.e. species) of the grid cell × species matrices (see above) were sorted according to their order on the phylogenetic trees using the ‘match.phylo.comm’ function of the R package ‘picante’ (Kembel et al. 2010). We identified evoregions (Maestri and Duarte, 2020; Nakamura et al. 2024) and phyloregions (Daru et al. 2016, 2020a, 2020b) for both fleas and hosts. Evoregion identification was based on the phylogenetic turnover among grid cells measured using the phylogenetic fuzzy-weighting method (Pillar and Duarte, 2010). This method accounts for both between-species phylogenetic distances and the imbalance in the numbers of tips descending from internal nodes (Duarte et al. 2016). The details of the methodology can be found in Maestri and Duarte (2020), and of its R implementation in Nakamura et al. (2024). In brief, the method starts with the construction of 2 matrices: a matrix Q of pairwise phylogenetic covariances between species standardized by marginal totals (showing the degree of the phylogenetic relationship of each species to every other species) and a presence/absence grid cell × species matrix. Then, these matrices are multiplied, resulting in a matrix P that represents the phylogenetic composition of species assemblages in each grid cell. The matrix P thus reflects the difference in the phylogenetic composition between cells (i.e. phylogenetic turnover), being a phylogenetic fuzzy matrix showing the phylogenetically weighted degree to which each species belongs to the assemblage of a given cell. This matrix (P) is then used as an input to calculate the Principal Coordinates of Phylogenetic Structure (PCPS; see Duarte, 2011), which involves a Principal Coordinate Analysis of matrix P, using square-rooted Bray-Curtis dissimilarities between cells to avoid negative eigenvalues. The eigenvectors of PCPS reflect the gradients of phylogenetic turnover across grid cells (Duarte et al. 2016; Maestri and Duarte, 2021). Subsequently, PCPS eigenvectors explaining more than 5% of variance are used as input data for the Discriminant Analysis of Principal Components based on k-means non-hierarchical clustering (DAPC; Jombart et al. 2010), which essentially performs regionalization. The resulting evoregions were shown to contain assemblages with higher phylogenetic cohesiveness/endemism than other metrics to calculate bioregions, such as the Simpson index of phylogenetic beta diversity (Maestri and Duarte, 2020). This procedure is implemented in the ‘calc_evoregion’ function of the R package ‘Herodotools’ (Nakamura et al. 2024).

The procedure to identify phyloregions is also based on the phylogenetic turnover of assemblages across grid cells (Daru et al. 2016). In contrast to the evoregion methodology, phylogenetic turnover for the phyloregion identification is calculated as the Simpson phylogenetic beta diversity index (phylogenetic βsim or pβsim, ranging from 0 to 1) (Daru et al. 2016). The advantage of the Simpson metric for turnover among assemblages is that it has been proven to be independent of differences in the number of species between sites (i.e. grid cells) (Koleff et al. 2003; Baselga and Leprieur, 2015). We calculated phylobeta diversity using the ‘phylobeta’ function of the R package ‘phyloregion’ (Daru et al. 2020b). Then, spatial clusters (i.e. phyloregions) were identified using one of the hierarchical clustering algorithms on the pβsim matrix and the optimal number of clusters. We identified phyloregions of either fleas or hosts using the ‘phyloregion’ function of the ‘phyloregion’ package using the default method, Unweighted Pair Group Means Algorithm (UPGMA).

Specifying the optimal number of clusters is necessary for clustering methods in both the ‘calc_evoregion’ and the ‘phyloregion’ functions to identify evoregions and phyloregions, respectively. In both the evoregion and phyloregion methodologies, the optimal number of clusters obtained from either DAPC (for evoregions) or UPGMA (for phyloregions) is determined by the same ‘elbow’ method, that is, selecting the smallest number of clusters that account for the largest proportion of explained variance in the data. For the evoregion identification, this number is automatically calculated in the ‘calc_evoregion’ function of the ‘Herodotools’ package using the embedded ‘optimal_phyloregion’ function from the ‘phyloregion’ package. In contrast, the ‘optimal_phyloregion’ function is used prior to the ‘phyloregion’ function for phyloregion identification. We selected the optimal number of phyloregions for both fleas and hosts that explained at least 90% of the variation.

After evoregions and phyloregions were identified, we aimed to understand whether a particular phylogenetic lineage of fleas or hosts predominantly occurred within particular evoregions or phyloregions. For this, we calculated species affiliations with evoregions or phyloregions, using the ‘calc_spp_association_evoreg’ function of ‘Herodotools’. Because many species occurred in more than 1 region, a species was considered to belong to an evoregion or a phyloregion if 55% of its occurrence grid cells were classified within that specific evoregion or phyloregion, respectively. Species that could not be affiliated with a single evoregion or phyloregion were considered to be widespread species (Maestri and Duarte, 2020). Then, we estimated ancestral evoregions and phyloregions simulating stochastic character (evoregion-specific/phyloregion-specific affiliation or being widespread) maps on a phylogenetic tree of either fleas or hosts using the ‘phytools’ package.

Next, we followed Ruggiero and Morrone (2025) and used the phylogenetic correspondence analysis (Pavoine, 2016) to identify key species or lineages that contributed most strongly to the observed evoregion or phyloregion differences. This method is an extension of the traditional correspondence analysis that replaces species with the units of a phylogenetic tree’s branch lengths, thus incorporating phylogenetic relationships and allowing the simultaneous analysis of the distributions of lineages among sites (in our case, evoregions or phyloregions) and the phylogenetic composition of these sites. The method builds ordination space, placing lineages and sites according to phylogenetic structure and phylogenetic composition, respectively. The phylogenetic correspondence analysis was carried out with the ‘evoCA’ function of the R package ‘adiv’ (Pavoine 2020, 2024).

Finally, we tested for the congruence between (a) evoregions or phyloregions identified for fleas and evoregions or phyloregions, respectively, identified for their hosts; (b) evoregions and phyloregions identified for either fleas or hosts; and (c) evoregions or phyloregions identified for either fleas or hosts and each of the regionalization schemes of Mongolia (main region physiographic, subregion physiographic, main biomes and ecoregions; see above). For each comparison, the congruence was assessed by the overall similarity of the regionalization’s spatial structure. This was done using the V-measure (Nowosad and Stepinski, 2018), which compares 2 regionalization maps and aims to quantitatively assess their spatial concordance. The V-measure is derived from a measure used in computer science for comparing different clusterings of the same domain and represents a harmonic mean of 2 metrics, namely homogeneity and completeness (see details in Nowosad and Stepinski, 2018). Homogeneity is the average homogeneity of the polygons of 1 map (e.g. Map 1) with respect to the polygons of another map (e.g. Map 2), whereas completeness is the average homogeneity of the polygons of Map 2 with respect to the polygons of Map 1. The V-measure ranges from 0 to 1 (from no congruence whatsoever to perfect congruence) and was calculated using the ‘vmeasure_calc’ function of the R package ‘sabre’ (Nowosad and Stepinski, 2018). Direct calculations of the V-measure do not contain testing for significance. To estimate significance of the V-measure values (i.e. whether congruence between 2 regionalization schemes differed from what would be expected by chance), we applied a randomization approach developed by Falaschi et al. (2023). For each 2 regionalizations (say, A and B), we calculated the V-measure values for the comparison of (a) an observed regionalization A with 999 randomly created regionalizations of regionalization B and (b) an observed regionalization B with 999 randomly created regionalizations of regionalization A, For each of these 2 steps, a *P* value representing the proportion of random comparisons showing a V-measure value higher (or lower) than the observed regionalization. Thus, each pairwise comparison provided 2 *P* values (one for comparison between regionalization A and randomized regionalization B and another for comparison between regionalization B and randomized regionalization A). We considered the congruence between 2 regionalizations schemes to be significant if both *P* values were <0.05. In addition, we followed Wang et al. (2024) and calculated the standardized effect size (SES) for each pairwise comparison as the difference between the observed V-measure and mean V-measure of randomized (=null) model divided by the standard deviation of the latter.

## Results

**Figure 1.**
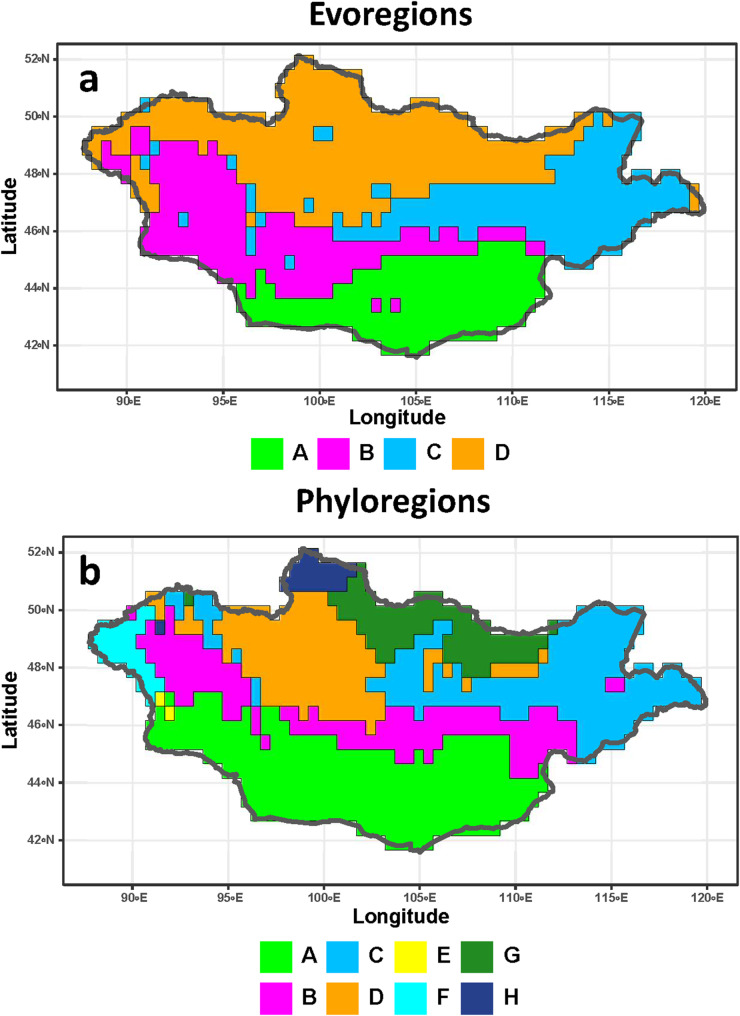
Evoregions (a) and phyloregions (b) for 66 species of fleas in Mongolia. Different evoregions and phyloregions are denoted by different colours.

**Figure 2.**
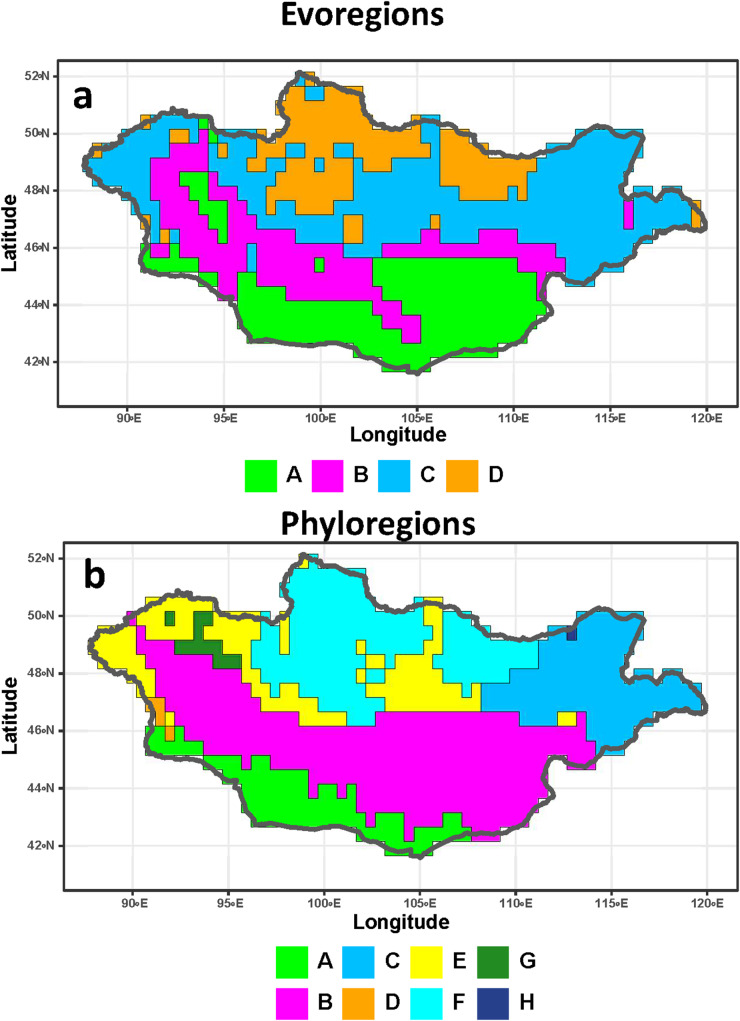
Evoregions (a) and phyloregions (b) for 64 rodent species parasitized by fleas in Mongolia. Different evoregions and phyloregions are denoted by different colours.

Four evoregions and 8 phyloregions for Mongolian fleas were identified (Figure 1). The same numbers of evoregions and phyloregions were identified for their hosts (Figure 2). In both fleas and hosts, evoregion A covered the southern part of Mongolia, whereas evoregion D covered its northern part. Evoregions B and C were situated to the north of evoregion A and to the south of evoregion D and stretched from eastern to western Mongolia. In addition, some flea lineages from evoregion B also occurred in evoregion A, whereas some lineages from evoregion C also occurred in evoregions B and D (Figure 1a). Flea evoregions A, B and C covered almost the same geographic extent as phyloregions A, B and C, whereas evoregion D corresponded to combined phyloregions D–H (Figure 1b). Distribution of the host evoregions was, to a certain degree, similar to that of fleas, although host evoregions A and D were roughly divided into 2 parts, with the southernmost portion of B intersecting the central part of A and the northernmost portion of C intersecting the central part of D (Figure 1a and Figure 2a). Similar to fleas, host evoregions A, B, and part of evoregion C covered almost the same areas with the respective phyloregions, but evoregion D covered approximately the same geographic extent as combined phyloregions E, G and F (Figure 2b). Furthermore, the geographic distribution of some phyloregions (A, B and C) was similar in fleas and hosts (Figure 1b and Figure 2b) but that of other phyloregions differed between them. In addition, the areas of 1 phyloregion of fleas (E) and 2 phyloregions of hosts (D and H) were very small and covered only a few grid cells (Figure 1b and Figure 2b, respectively).

**Figure 3.**
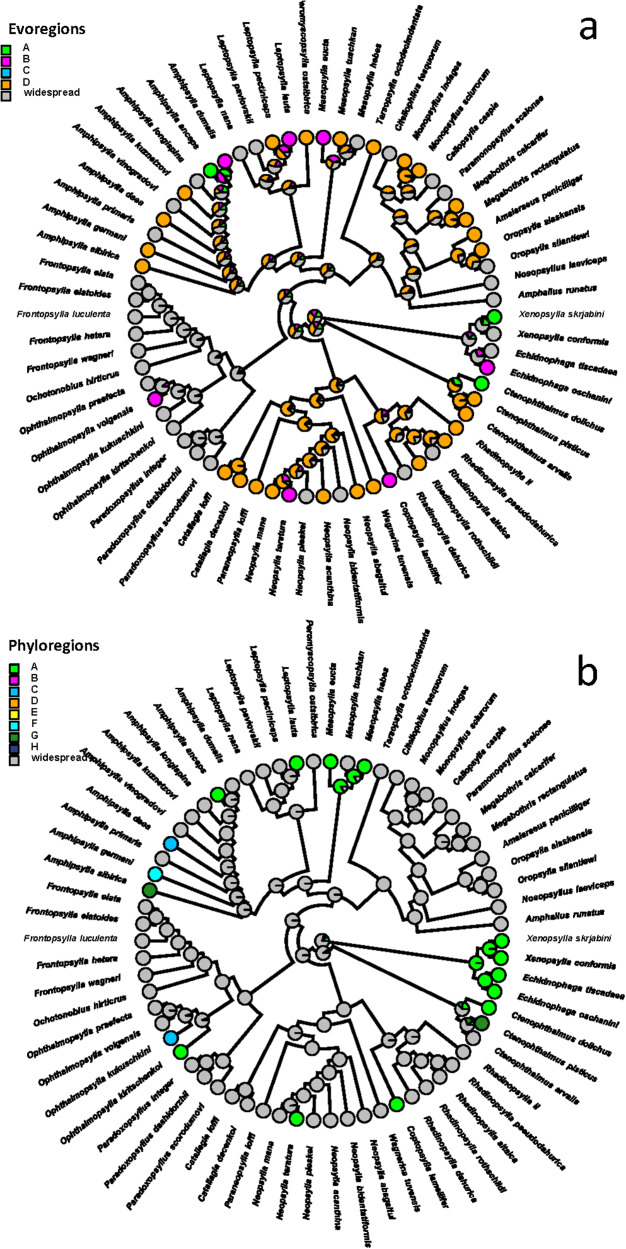
Phylogenetic tree of fleas parasitic on rodents in Mongolia with colours representing the predominant evoregions (a) and phyloregions (b) (at least 55% of a species’ geographic range belongs to a given region). Widespread species are those in which 55% of their geographic range could not be attributed to a single evoregion or phyloregion.

The reconstruction of ancestral evoregions suggested that most flea lineages were mainly affiliated with evoregion C (e.g. the northern and central parts of Mongolia) and dispersed from it to other evoregions (Figure 3a). Regarding phylogenetic distinctness (i.e. distribution among phyloregions), many flea species and lineages appeared to occur in multiple phyloregions, except for Pulicidae (genera *Xenopsylla* and *Echidnophaga*) and the leptopsyllid genus *Mesopsylla*, which were clearly affiliated with phyloregion A (southern Mongolia) (Figure 3b).

**Figure 4.**
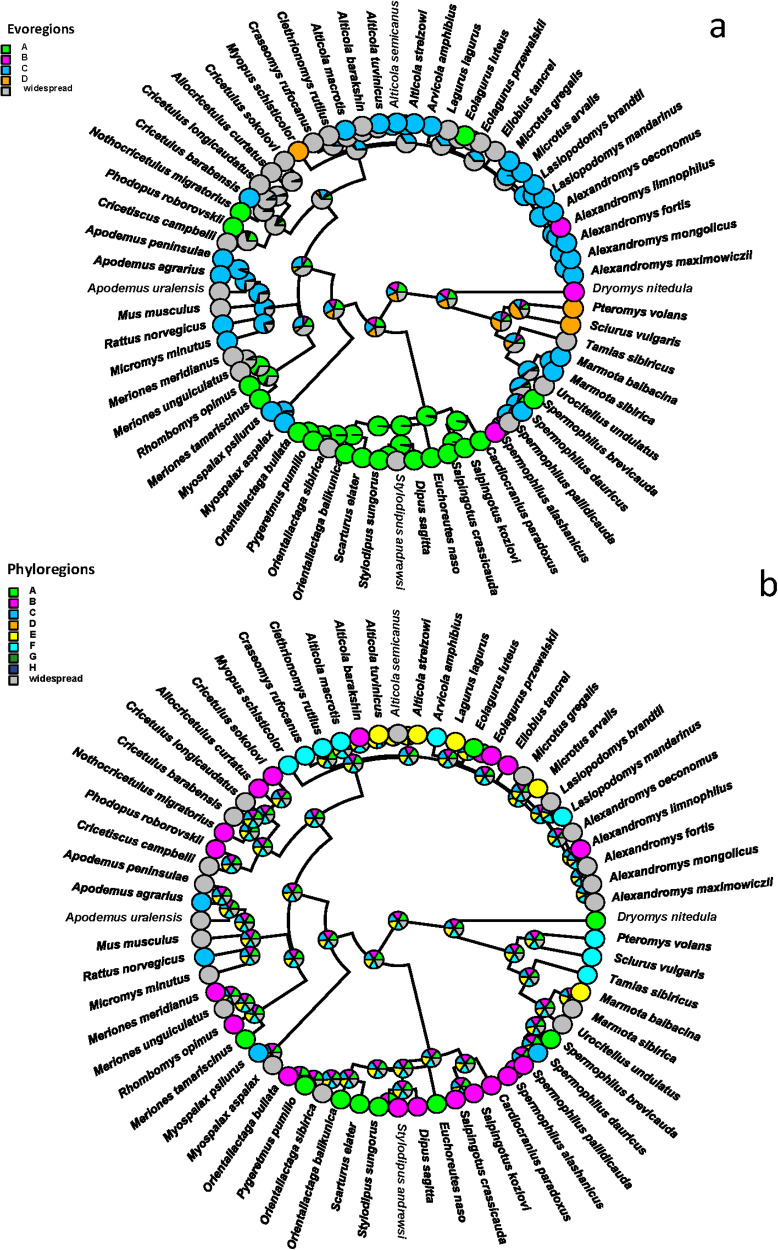
Phylogenetic tree of Mongolian rodents harbouring at least 1 of 66 flea species for which evoregion and phyloregion regionalizations were carried out, with colours representing the predominant evoregions (a) and phyloregions (b) (at least 55% of a species’ geographic range belongs to a given region). Widespread species are those in which 55% of their geographic range could not be attributed to a single evoregion or phyloregion. Note that *Dipus sagitta, Meriones meridianus, Alexandromys mongolicus* and *Ellobius tancrei* each likely represent a complex of very closely related species (Lisenkova et al. 2023; Nanova et al. 2020; Lissovsky et al. 2018; Lebedev et al. 2020, respectively).

Affiliations of host lineages with evoregions were more pronounced than those with phyloregions (Figure 4). In particular, dipodid lineages were characteristic of evoregion A, whereas most microtines, some marmotines and murines were found in evoregion D (Figure 4a). The phylogenetic distinctness of phyloregions appeared to be mostly determined at the species level rather than at a deeper phylogenetic level, with different species belonging to different lineages occurring within a given phyloregion (Figure 4b). Again, southern Mongolia (phyloregion A) was characterized by many dipodid species, whereas species from other lineages were diffusely distributed across several phyloregions (Figure 4b).

**Figure 5.**
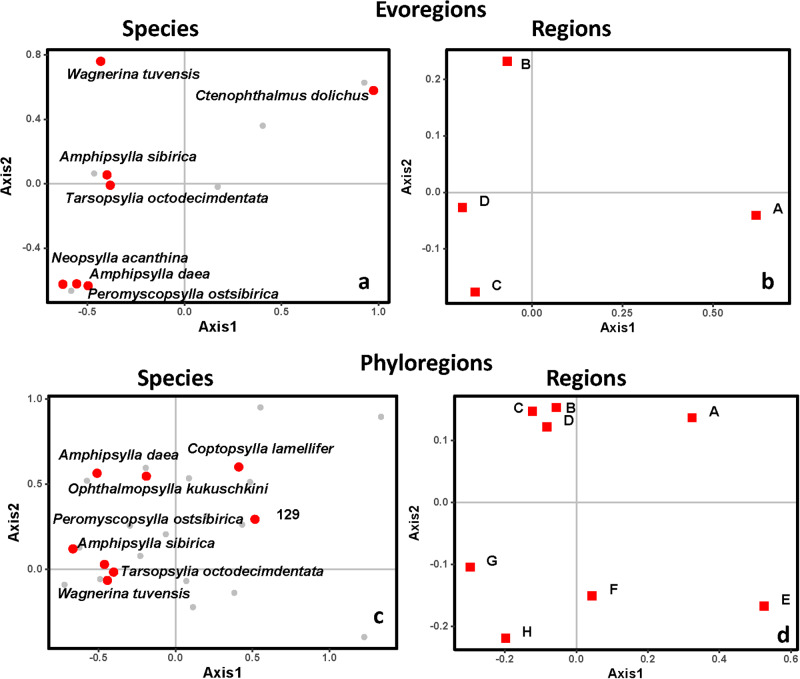
Ordination diagrams produced by phylogenetic correspondence analysis (evoCA) of the data on Mongolian fleas parasitic on rodents demonstrating the coordinates on the first 2 axes of evoCA for flea species (a, c) and either evoregions (b) or phyloregions (d). Species contributing to at least 5% of the explained variation in phylogenetic composition across evoregions or phyloregions are in red.

**Figure 6.**
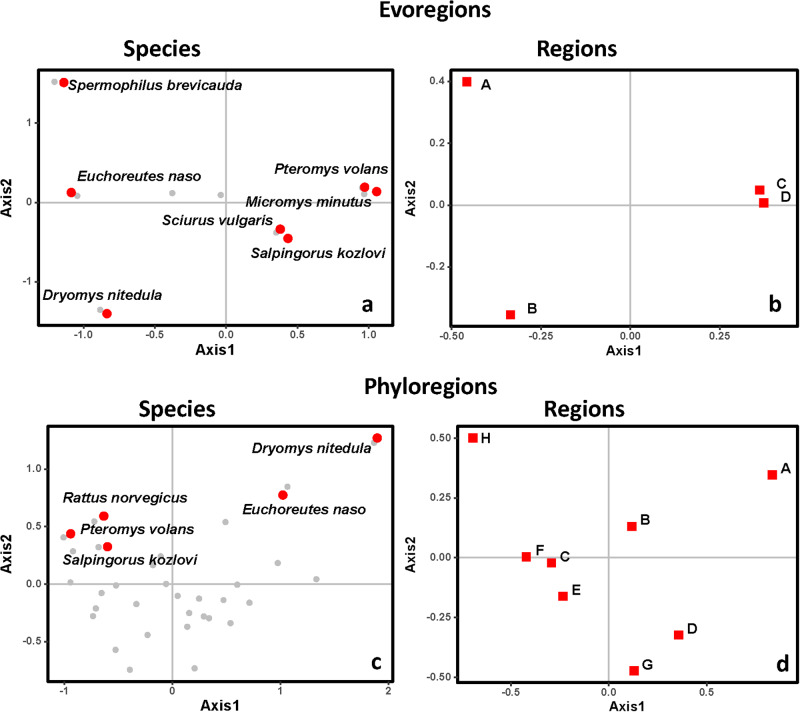
Ordination diagrams produced by phylogenetic correspondence analysis (evoCA) of the data on Mongolian rodents harbouring at least 1 of 66 flea species demonstrating the coordinates on the first 2 axes of evoCA for rodent species (a, c) and either evoregions (b) or phyloregions (d). Species contributing to at least 5% of the explained variation in phylogenetic composition across evoregions or phyloregions are in red.

Phylogenetic correspondence analyses revealed flea and host species that contributed the most to the differences in the respective phylogenetic compositions of evoregions and phyloregions. Five of 7 flea species that mainly contributed to the phylogenetic differences between evoregions also contributed the most to the phylogenetic differences between phyloregions (Figures 5a, 5c). Similarly, 4 of 7 main host contributors to the phylogenetic differences between evoregions were also contributors to the phylogenetic differences between phyloregions (Figures 6a, 6c). However, despite the geographic overlap between some evoregions and phyloregions for both fleas and hosts (see above), phylogenetic differences between the former and the latter were determined by different species (Figures 5b, 5d for and Figures 6b, 6d, respectively).

The degrees of spatial association between regionalizations assessed via the V-measure are presented in Table 1. For the majority of regionalization comparisons, the observed congruence between regionalizations differed significantly from expected by chance (see SES values for V-measure in Tables S1 and S2 in Appendix 2 of the Supplementary Material). For both fleas and hosts, subdivisions of Mongolia into evo- and phyloregions demonstrated moderate similarity. The same was the case when the geographic distributions of evo- and phyloregions were compared between fleas and hosts, although the similarity between flea and host phyloregionalizations was slightly higher than that for evoregionalizations. The congruence of evoregions with the main region physiography of Mongolia was higher for evo- than for phyloregions in fleas, but the opposite was true for hosts. In both fleas and hosts, the subregion physiography and geographic distributions of ecoregions were more similar for phylo- than for evoregions, although similarity between subregion physiography appeared to be random for flea phyloregions and host evo- and phyloregions (Table 1, Tables S1–S2 of Appendix 2, Supplementary Material). Finally, the similarity with ecoregions was higher for flea phylo- than for flea evoregions, whereas the congruence between ecoregions and either host evo- or phyloregions demonstrated the opposite trend. Comparing fleas and hosts, it can be seen that the flea evo- and phyloregions tended to show higher congruence in main region physiography, biomes and ecoregions, while the host evo- and phyloregions had lower correspondence with the same environmental schemes (although not with subregion physiography).

**Table 1.** Congruence between regionalizations measured via the V-measure (V), homogeneity (H) and completeness (C)

Comparison of regionalizations	V	H	C
F: between evoR and phyloR	0.50*	0.56	0.45
H: between evoR and phyloR	0.49*	0.54	0.45
F: between evoR and main region physiography	0.49*	0.49	0.50
H: between evoR and main region physiography	0.40*	0.40	0.41
F: between phyloR and main region physiography	0.46*	0.41	0.53
H: between phyloR and main region physiography	0.43*	0.39	0.48
F: between evoR and subregion physiography	0.45*	0.60	0.36
H: between evoR and subregion physiography	0.39	0.52	0.32
F: between phyloR and subregion physiography	0.48	0.56	0.43
H: between phyloR and subregion physiography	0.51	0.60	0.44
F: between evoR and biomes	0.38*	0.35	0.40
H: between evoR and biomes	0.37	0.34	0.39
F: between phyloR and biomes	0.40*	0.44	0.49
H: between phyloR and biomes	0.35	0.30	0.41
F: between evoR and ecoregions	0.44*	0.60	0.35
H: Between evoR and ecoregions	0.38*	0.53	0.50
F: between phyloR and ecoregions	0.50*	0.59	0.43
H: between phyoR and ecoregions	0.46*	0.57	0.39
EvoR between F and H	0.50*	0.49	0.50
PhyloR between F and H	0.53*	0.52	0.55

EvoR, evoregions; PhyloR, phyloregions; F, fleas; H, hosts. Asterisks denote significant similarity between regionalizations (significantly different from what is expected by chance; both A-random B and B-random A. *P* values <0.05; see text for explanations).

## Discussion

Biogeographic regionalization serves as a fundamental framework to understand the interplay of the historical processes that shape species distributions and biodiversity patterns (e.g. Kreft and Jetz, 2010; Ficetola et al. 2017; Morrone, 2018; Bernardo-Madrid et al. 2025). By applying advanced phylogenetic regionalization methods to a host-parasite system in Mongolia, our study offers insights into how *in situ* diversification and *ex situ* dispersal processes can manifest at regional scales and how these patterns align across interacting taxa and with environmental gradients. We found that different phylogenetic regionalization approaches capture distinct evolutionary signals. Our findings highlight that evoregions and phyloregions, even for the same taxon, delineate spatially distinct and evolutionarily unique areas. For both fleas and hosts, we identified 4 evoregions and 8 phyloregions. This divergence in the number and spatial extent of regions underscores the fundamental differences between these methods. Evoregionalization, which emphasizes the balance between *in situ* diversification and *ex situ* dispersal (Maestri and Duarte, 2020; Nakamura et al. 2024), appears to aggregate phylogenetic signals more broadly. In contrast, phyloregionalization, based on overall phylogenetic turnover, yields a finer-scale partitioning of phylogenetic distinctness. The phylogenetic correspondence analyses reinforced this distinction: while some key species contributed to differences in both evoregions and phyloregions, the overall phylogenetic differences defining evoregions versus phyloregions for a given taxon were driven by distinct sets of species. This finding suggests that even when spatial boundaries show overlap, the underlying evolutionary processes defining these boundaries may differ between the 2 methods. Therefore, researchers must carefully consider their specific hypotheses regarding diversification versus dispersal when choosing a phylogenetic regionalization approach, as each method can illuminate different facets of evolutionary history.

Our study also sheds light on the aspect of scale-dependence in biogeographic congruence. Previous work at a much larger scale (the entire Palearctic) revealed a significant mismatch between the evoregions of fleas and their hosts (Krasnov and Shenbrot, 2023), with the V-measure, which reflects spatial congruence between flea and host evoregions, being 0.33. In contrast, our analyses at the regional scale of Mongolia demonstrated a substantially higher (albeit not especially high) spatial similarity between flea and host regionalizations (V-measure = 0.49–0.50). This scale-dependent congruence suggests that at broader geographical extents, deep evolutionary divergences and unique macroevolutionary histories may lead to independent biogeographic structuring of hosts and their parasites. However, at a regional scale, more recent historical events, perhaps shared dispersal routes and contemporary ecological interactions likely contribute to a greater degree of co-regionalization. The slightly higher congruence in phyloregions might indicate that shared dispersal or environmental filtering events that influence species turnover are likely more synchronous between hosts and parasites at this regional scale than the deeper diversification patterns captured by evoregions.

Although evoregions and phyloregions do not fully align, there are, nevertheless, coarse-scale similarities, especially in the southern bioregions (evo- and phyloregions A and B), the northern bioregions (evoregion D and phyloregions D, G and H for fleas; evoregion D and phyloregions E and F for hosts; Figures 1 and 2), and the eastern bioregions (evoregion and phyloregion C for fleas and partly for hosts). Evoregions seem to emphasize deeper clade turnover, generating some high phylogenetic affinities between hosts and evoregions, but much less so for fleas, possibly reflecting more coherent diversification events or regional endemism in host clades. This suggests high phylogenetic turnover among the evoregions, especially for hosts. Phyloregions emphasized more terminal phylogenetic similarity, with most flea species, as well as many host species, designated as widespread. The widespread occurrence of many species suggests that phylogenetic turnover is spatially diffused, albeit high. The presence of ‘widespread’ species/clades suggests that strict endemism is not universal, reducing the correspondence between species distributions and regionalizations.

Still, some regions of endemism stood out. For instance, southern Mongolia, where evoregion A is located, appeared to be a very different region from the others, both for fleas and, especially, for hosts (due to allactagine jerboas) (Figures 3 and 4). Phyloregionalization also detected this south bioregion as different from the others, especially for fleas (due to pulicids) (Figure 3). The distinct position of southern Mongolia, from the perspective of phylogenetic endemism, can also be seen along the first axis of the phylogenetic correspondence analysis, albeit in slightly different positions for fleas and hosts (evoregion A for fleas and phyloregion A for hosts; Figures 5 and 6). At the other extreme, the northern evo- and phyloregions differed from the others for fleas. Overall, it seems there is a dominant north-south gradient, followed by an east-to-west gradient, that explains most of the phylogenetic composition and, therefore, the geographic distribution of evo- and phyloregions.

The reconstructed ancestral evoregions suggested that the shaping of Mongolian flea faunas (or an original point of flea invasions into Mongolia) likely started in northern Mongolia (evoregion D, i.e. the border of the Siberian province of the Euro-Siberian subregion and the Central Asian subregion of the Palearctic; Medvedev, 2014; Medvedev et al. 2017). From there, fleas dispersed outwards, possibly via the corridor between the Khangai and the Khentii Mountains. We recognize that this scenario is highly speculative and requires further investigation; nevertheless, it further points to dispersal as a significant driver shaping the fleas’ regional patterns, potentially leading to increased co-occurrence with hosts.

The interactions between hosts and parasites, particularly regarding their co-regionalization, present an interesting dimension for biogeographers. Our findings of moderate congruence between flea and host regionalizations in Mongolia, yet differences in their specific environmental associations, suggest a dynamic interaction. Imago fleas are obligatory haematophagous parasites, utterly dependent on their hosts. On the contrary, pre-imagoes of the absolute majority of fleas species are not parasitic, with the larvae feeding on all kinds of organic matter found in the burrows or nests of their hosts (e.g. Marshall, 1981). Nevertheless, flea regionalization patterns appeared to be not simply a passive reflection of host biogeography. Moreover, the generally higher degrees of congruence between flea evo- and phyloregions and physiographic and ecological schemes, in comparison to those of hosts, are particularly interesting. These counter-intuitive results may indicate that despite fleas being ultimately host-dependent, their distributions may be more strongly influenced by specific environmental conditions or habitat characteristics within these broad zones than those of their hosts. Indeed, flea distribution has been shown to depend almost equally on both host identities and environmental conditions. This appeared to be the case at local (e.g. Krasnov et al. 1997), regional (e.g. Baláž et al. 2021) and global (e.g. Krasnov et al. 2020) scales. Furthermore, fleas, especially pre-imagoes, are characterized by extremely narrow tolerance to environmental factors such as air temperature, relative humidity and soil structure (Krasnov et al. 2001a, 2001b, 2002a, 2002b; van der Mescht et al. 2016). Hosts, on the other hand, may tolerate a broader range of conditions; alternatively, their dispersal capacities, undoubtedly higher than those of fleas, may lead to broader distribution ranges that blur finer environmental boundaries, as supported by their lower correspondence with some environmental schemes.

In conclusion, our study underscores the power of integrating phylogenetic information into biogeographic regionalization. It demonstrates that evoregionalization and phyloregionalization provide complementary perspectives on the evolutionary processes that shape regional biodiversity patterns. The distinct patterns observed for fleas and their hosts in Mongolia, coupled with the scale-dependent congruence of their regionalizations, provide compelling evidence for the complex interplay between host-parasite interactions, deep evolutionary history and contemporary environmental drivers. For biogeographers working across any taxa or regions, our findings emphasize the need to: (a) carefully select phylogenetic regionalization methods based on the specific evolutionary processes and scale under investigation; (b) acknowledge the influence of spatial scale on the observed biogeographic congruence; and (c) recognize the intricate, often non-linear, relationships between interacting species’ regionalizations and their environmental contexts.

## Supporting information

10.1017/S0031182025101212.sm001 Maestri et al. supplementary material Maestri et al. supplementary material

## Data Availability

Raw data on flea and host species distribution across Mongolia can be obtained from the corresponding author upon request.

## Supplementary material

The supplementary material for this article can be found at https://doi.org/10.1017/S0031182025101212.
