# Supplementary material for: Phylogenetic regionalization of ectoparasites and their hosts using 2 approaches: a case study with fleas and their rodent hosts from Mongolia
Source: Parasitology. 2025 Nov 21;153(1):89–102. doi: 10.1017/S0031182025101212 (PMC13215760; doi:10.1017/S0031182025101212)
Supplement: Maestri et al. supplementary material [file S0031182025101212sup001.docx]

**Appendix 1. Supplementary Figures**


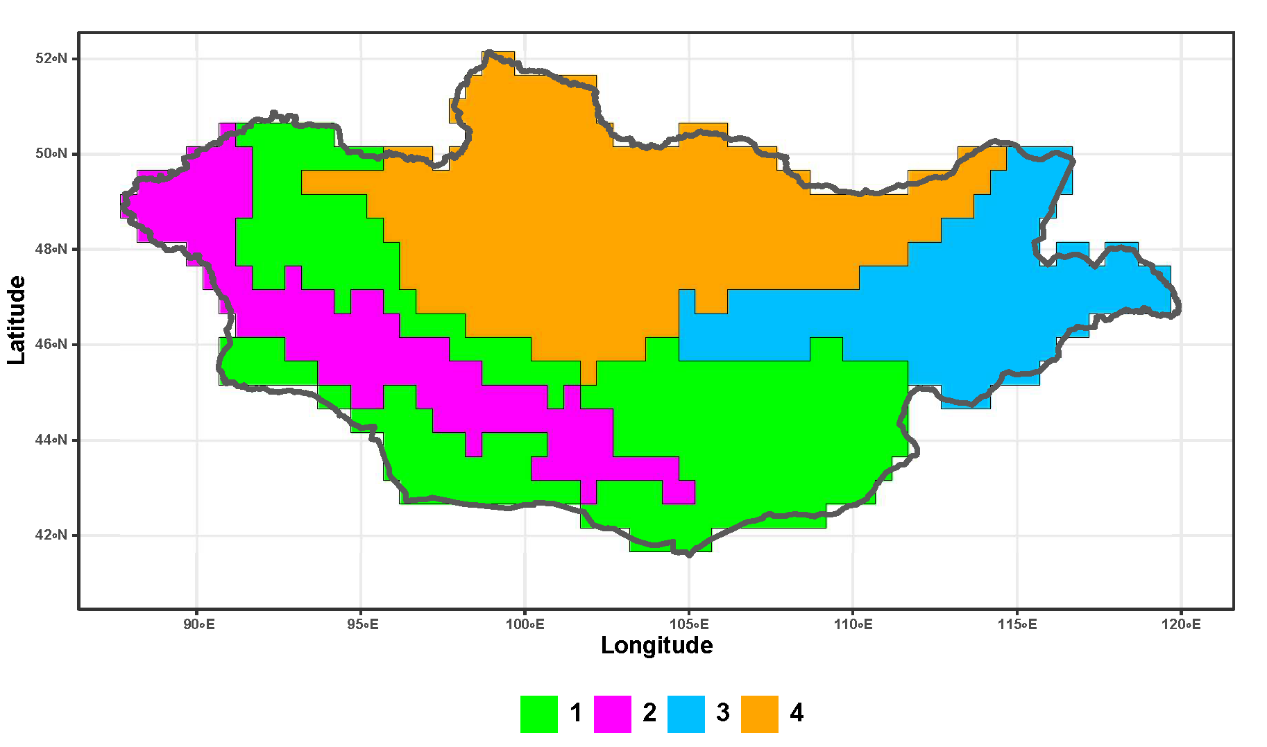


**Supporting Figure S1** Map of the main physiographic regions of Mongolia (0.5° × 0.5° grid). 1: main Gobi region, 2: main Altai Mountains region, 3: main Mongolian Eastern Plain region, 4: main Khangai-Khentii Mountains region.


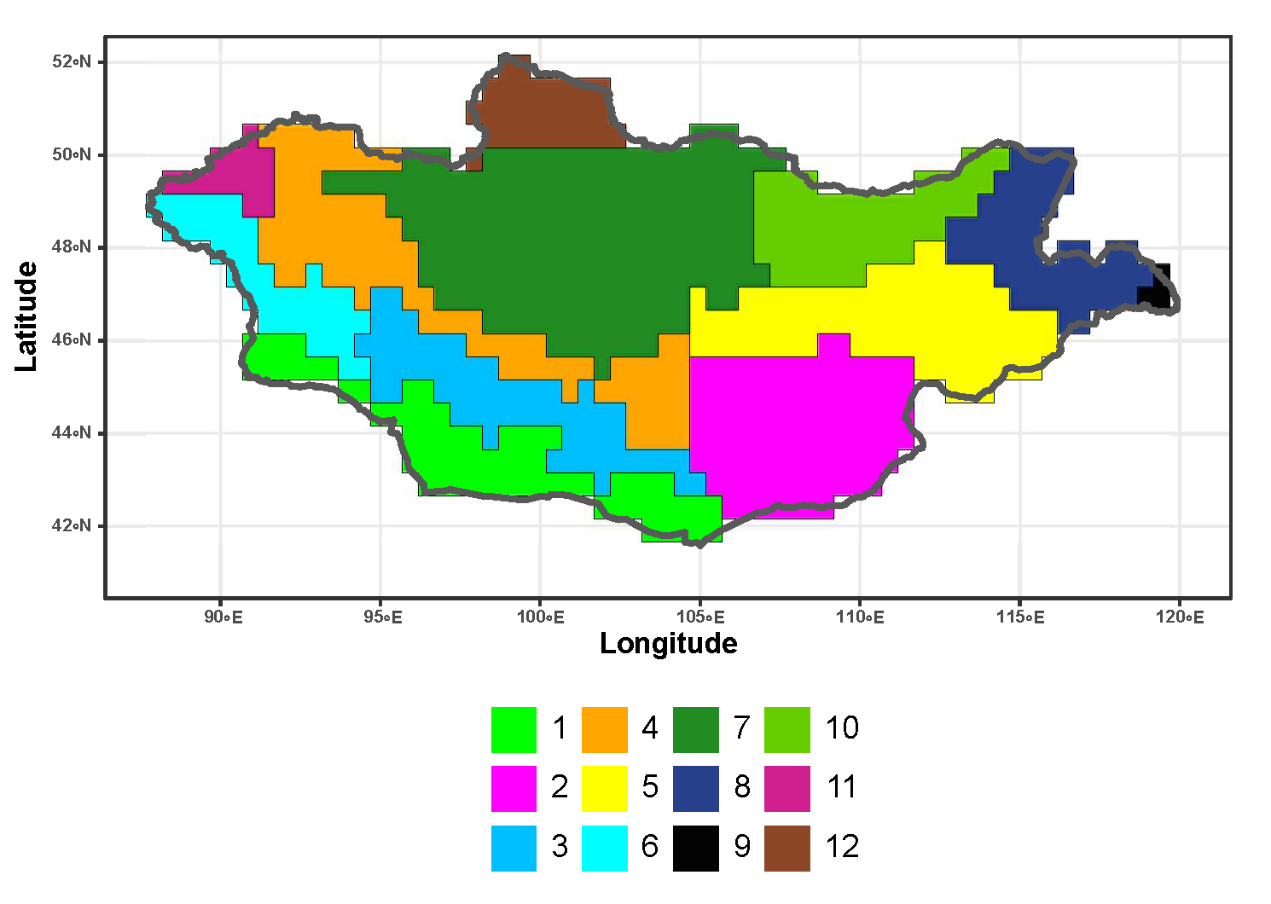


**Supporting Figure S2** Map of physiographic subregions of Mongolia (0.5° × 0.5° grid). 1: Trans-Altai Gobi, 2: Eastern Gobi, 3: Gobi Altai, 4: Gobi region of Northern Altai, 5: Middle Khalkh and Dariganga plain, 6: Mongol Altai, 7: Khangai, 8: Eastern plain, 9: Khyangan, 10: Khentii, 11: Siikhem-Kharkhiraa, 12: Khovsgol.


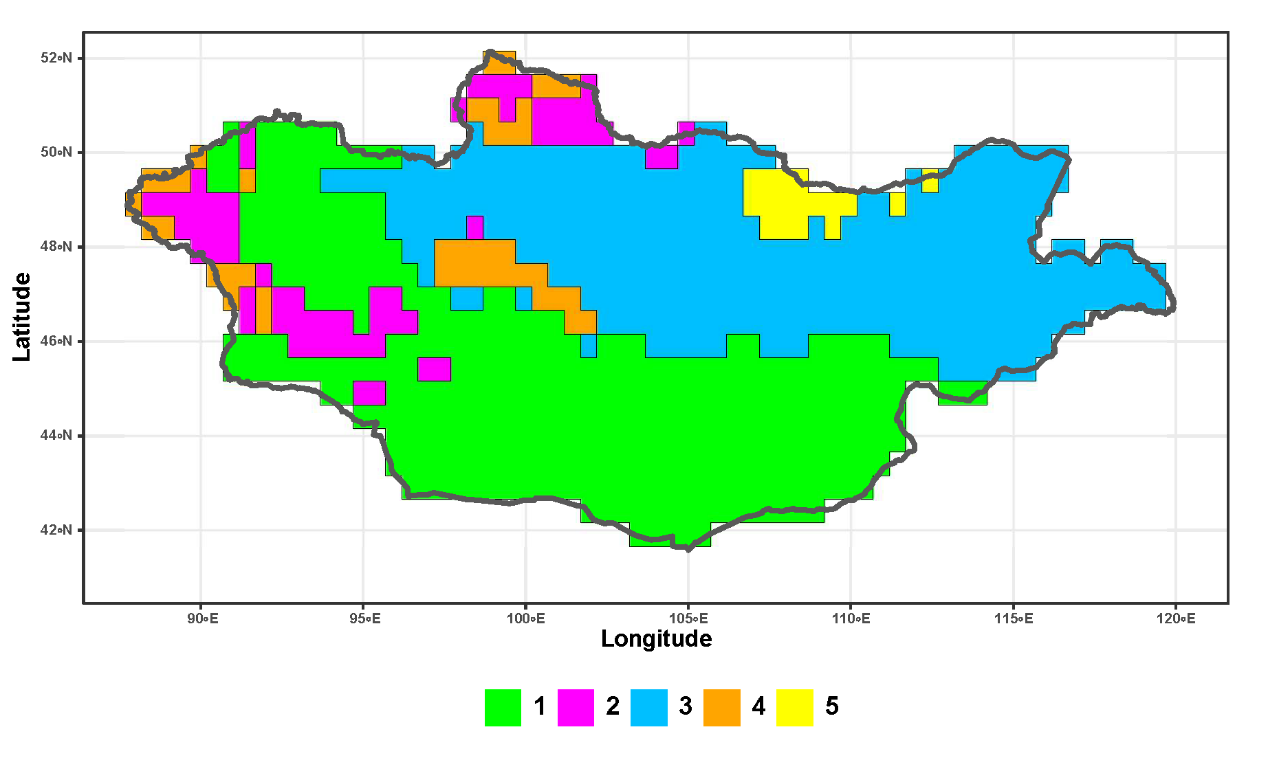


**Supporting Figure S3** Map of main biomes of Mongolia (0.5° × 0.5° grid). 1: deserts and xeric shrublands, 2: temperate conifer forests, 3: temperate grasslands and shrublands, 4: montane grasslands and shrublands, 5: boreal forests/taiga.

**Supporting Figure S4** Map of ecoregions of Mongolia (0.5° × 0.5° grid). 1: Alashan Plateau semi-desert, 2: Eastern Gobi desert steppe, 3: Junggar Basin semi-desert, 4: Gobi Lakes Valley desert steppe, 5: Altai montane forest and forest steppe, 6: Mongolian-Manchurian grassland, 7: Selenge-Orkhon forest steppe, 8: Altai alpine meadow and tundra, 9: Great Lakes Basin desert steppe, 10: Khangai Mountains alpine meadow, 11: Daurian forest steppe, 12: Khangai Mountains conifer forests, 13: Trans-Baikal conifer forest, 14: Sayan alpine meadows and tundra, 15: Sayan montane conifer forests


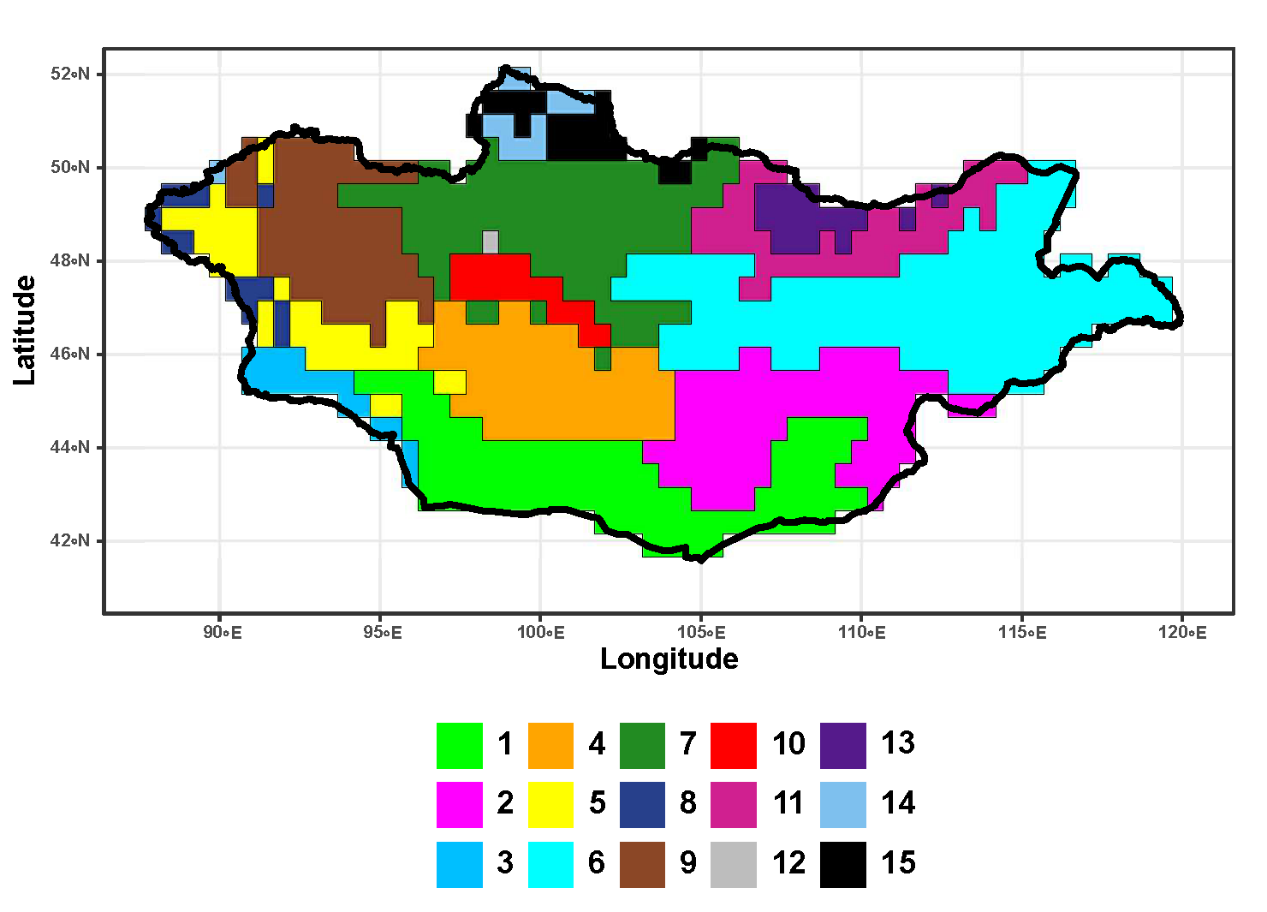


**Appendix 2. Supplementary Tables**

**Supporting Table S1** Standardized effect sizes (SES) of the V-measure calculated as differences between the observed V-measure of regionalization A and mean V-measure obtained from randomized regionalizations of regionalization B divided by the standard deviation randomized regionalizations of regionalization B. Regionalizations A are in columns, regionalizations B are in rows. EvoR: evoregions, PhyloR: phyloregions, F: fleas, H: hosts, MRPh – main physiographic regionalization, SRph – subregiona physiographic regionalization, Biomes – regionalization by main biomes, Ecoregions – regionalization by ecoregions.

|  | F:EvoR | H:EvoR | F:PhyloR | H:PhyloR | MRPh | SRPh | Biomes | Ecoregions |
| --- | --- | --- | --- | --- | --- | --- | --- | --- |
| F:EvoR | - | 6.16 | 5.05 | - | 3.73 | 1.14 | 3.24 | 4.56 |
| H:EvoR | 4.15 | - | - | 4.33 | 2.24 | -0.10 | 3.07 | 3.41 |
| F:PhyloR | 4.21 | - | - | 4.62 | 2.87 | -0.15 | 3.09 | 6.25 |
| H:PhyloR | - | 6.62 | 5.24 | - | 2.16 | 0.74 | 1.79 | 5.16 |
| MRPh | 4.06 | 4.43 | 4.18 | 2.95 | - | - | - | - |
| SRPh | 3.75 | 4.57 | 3.79 | 4.91 | - | - | - | - |
| Biomes | 1.80 | 3.72 | 2.29 | 0.56 | - | - | - | - |
| Ecoregions | 3.15 | 4.10 | 4.87 | 3.33 | - | - | - | - |

**Supporting Table S2** Standardized effect sizes (SES) of the V-measure calculated as differences between the observed V-measure of regionalization B and mean V-measure obtained from randomized regionalizations of regionalization A divided by the standard deviation randomized regionalizations of regionalization A. Regionalizations B are in columns, regionalizations A are in rows. EvoR: evoregions, PhyloR: phyloregions, F: fleas, H: hosts, MRPh – main physiographic regionalization, SRph – subregiona physiographic regionalization, Biomes – regionalization by main biomes, Ecoregions – regionalization by ecoregions.

|  | F:EvoR | H:EvoR | F:PhyloR | H:PhyloR | MRPh | SRPh | Biomes | Ecoregions |
| --- | --- | --- | --- | --- | --- | --- | --- | --- |
| F:EvoR | - | 4.15 | 5.05 | - | 4.06 | 3.75 | 1.80 | 3.15 |
| H:EvoR | 5.05 | - | - | 6.62 | 4.43 | 4.57 | 3.72 | 4.10 |
| F:PhyloR | 6.16 | - | - | 5.24 | 4.18 | 3.79 | 2.29 | 4.87 |
| H:PhyloR | - | 4.33 | 5.24 | - | 2.95 | 4.91 | 0.56 | 3.33 |
| MRPh | 3.73 | 2.24 | 4.18 | 2.16 | - | - | - | - |
| SRPh | 1.14 | -0.10 | 3.79 | 0.74 | - | - | - | - |
| Biomes | 3.24 | 3.07 | 2.29 | 1.79 | - | - | - | - |
| Ecoregions | 4.56 | 3.41 | 4.87 | 5.16 | - | - | - | - |
